# Supplementary material for: Diagnostic Accuracy of Various Immunochromatographic Tests for NS1 Antigen and IgM Antibodies Detection in Acute Dengue Virus Infection
Source: Int J Environ Res Public Health. 2022 Jul 19;19(14):8756. doi: 10.3390/ijerph19148756 (PMC9324781; doi:10.3390/ijerph19148756)
Supplement: Supplementary file 1 [file ijerph-19-08756-s001.zip › ijerph-1761799-supplementary.pdf]

Supplementary Table

Supplementary Table S1: Excluded Full-Text Studies (N = 36).

No.	Author, year	Title	DOI
1	Lima, 2010	Comparison of three commercially available dengue NS1 antigen capture assays for acute diagnosis of dengue in Brazil	https://doi.org/10.1371/journal.pntd.0000738
2	Mata, 2017	Accuracy and reliability of an NS1 rapid immunochromatographic test for DENV-1 diagnosis at point of care and in the laboratory	https://doi.org/10.1186/s12879-017-2679-z
3	Piedrahita, 2016	Evaluation of Commercially Available Assays for Diagnosis of Acute Dengue in Schoolchildren during an Epidemic Period in Medellin, Colombia	https://doi.org/10.4269%2Fajtmh.15-0492
4	Vega, 2009	Evaluation of PANBIO rapid immunochromatographic cassette for dengue diagnosis in a Colombian endemic area	https://pubmed.ncbi.nlm.nih.gov/20440460/
5	Simonnet, 2017	Prospective evaluation of the SD BIOLINE Dengue Duo rapid test during a dengue virus epidemic	https://doi.org/10.1007/s10096-017-3083-8
6	Vickers, 2017	Evaluation of OneStep Dengue NS1 RapiDip™ InstaTest and OneStep Dengue Fever IgG/IgM RapiCard™ InstaTest during the course of a dengue type 1 epidemic	https://doi.org/10.1016/j.diagmicrobio.2017.08.019
7	Vickers, 2015	The performance of the SD BIOLINE Dengue DUO® rapid immunochromatographic test kit for the detection of NS1 antigen, IgM and IgG antibodies during a dengue type 1 epidemic in Jamaica	https://doi.org/10.1186/s12929-015-0164-9
8	Sánchez-Vargas, 2014	Evaluation of the SD BIOLINE dengue duo rapid test in the course of acute and convalescent dengue infections in a Mexican endemic region	https://doi.org/10.1016/j.diagmicrobio.2013.12.019
9	Ramirez, 2009	Evaluation of dengue NS1 antigen detection tests with acute sera from patients infected with dengue virus in Venezuela	https://doi.org/10.1016/j.diagmicrobio.2009.07.022
10	Sharmin, 2011	Evaluation of an immunochromatographic test for early and rapid detection of dengue virus infection in the context of Bangladesh.	https://apps.who.int/iris/handle/10665/171008
11	Shukla, 2017	Utility of dengue NS1 antigen rapid diagnostic test for use in difficult to reach areas and its comparison with dengue NS1 ELISA and qRT-PCR	https://doi.org/10.1002/jmv.24764
12	Garg, 2019	Can rapid dengue diagnostic kits be trusted? A comparative study of commercially available rapid kits for serodiagnosis of dengue fever	https://doi.org/10.4103/jlp.jlp_140_18
13	Shrivastava, 2011	Evaluation of a commercial Dengue NS1 enzyme-linked immunosorbent assay for early diagnosis of dengue infection	https://doi.org/10.4103/0255-0857.76525
14	Sathish, 2001	Comparison of IgM capture ELISA with a commercial rapid immunochromatographic card test & IgM microwell ELISA for the detection of antibodies to dengue viruses	https://pubmed.ncbi.nlm.nih.gov/12138661/
15	Vajpayee, 2001	Comparative Evaluation of Various Commercial Assays for Diagnosis of Dengue Fever	https://pubmed.ncbi.nlm.nih.gov/11944701/
16	Babalish, 2006	Catching Dengue Early: Clinical Features and Laboratory Markers of Dengue Virus Infection	https://europepmc.org/article/med/26591143
17	Moorthy, 2009	Evaluation of a rapid immunochromatographic device for the detection of IgM & IgG antibodies to dengue viruses (DENV) in a tertiary care hospital in south India	https://doi.org/10.4103/0255-0857.53210
18	Gaikwad, 2017	Comparison of nonstructural protein-1 antigen detection by rapid and enzyme-linked immunosorbent assay test and its correlation with polymerase chain reaction for early diagnosis of dengue	https://doi.org/10.4103/0974-2727.208265
19	Blacksell, 2007	Prospective Study To Determine Accuracy of Rapid Serological Assays for Diagnosis of Acute Dengue Virus Infection in Laos	https://doi.org/10.1128/CVI.00482-06
20	Blessmann, 2020	Assessment of diagnostic and analytic performance of the SD Bioline Dengue Duo test for dengue virus (DENV) infections in an endemic area (Savannakhet province, Lao People's Democratic Republic)	https://doi.org/10.1371/journal.pone.0230337
21	Somlor, 2021	Evaluation of VIDAS® Diagnostic Assay Prototypes Detecting Dengue Virus NS1 Antigen and Anti-Dengue Virus IgM and IgG Antibodies	https://doi.org/10.3390/diagnostics11071228
22	Chong, 2020	Diagnostic accuracy and utility of three dengue diagnostic tests for the diagnosis of acute dengue infection in Malaysia	https://doi.org/10.1186/s12879-020-4911-5

23	Fry, 2011	The Diagnostic Sensitivity of Dengue Rapid Test Assays Is Significantly Enhanced by Using a Combined Antigen and Antibody Testing Approach	https://doi.org/10.1371/journal.pntd.0001199
24	Jusoh, 2017	Performance Evaluation of Commercial Dengue Diagnostic Tests for Early Detection of Dengue in Clinical Samples	https://doi.org/10.1155/2017/4687182
25	Fredolin, 2018	Evaluation of a commercial dengue combo rapid test kit for the detection of NS1 and IgM	https://pubmed.ncbi.nlm.nih.gov/33601826/
26	Teoh, 2016	The Use of NS1 Rapid Diagnostic Test and qRT-PCR to Complement IgM ELISA for Improved Dengue Diagnosis from Single Specimen	https://doi.org/10.1038/srep27663
27	Pun, 2012	Prognostic Value of Rapid Test for Diagnosis of Dengue in Nepalese Patients during 2010 Epidemic	https://doi.org/10.3126/kumj.v10i1.6905
28	Guzman, 2010	Dengue: a continuing global threat	https://doi.org/10.1038/nrmicro2460
29	Pal, 2015	Multicountry Prospective Clinical Evaluation of Two Enzyme-Linked Immunosorbent Assays and Two Rapid Diagnostic Tests for Diagnosing Dengue Fever	https://doi.org/10.1128/JCM.03042-14
30	Vaira, 2016	Clinical, Virologic, and Epidemiologic Characteristics of Dengue Outbreak, Dar es Salaam, Tanzania, 2014	https://doi.org/10.3201%2Feid2205.151462
31	Lim, 2019	Clinical and epidemiologic characteristics associated with dengue during and outside the 2016 outbreak identified in health facility-based surveillance in Ouagadougou, Burkina Faso	https://doi.org/10.1371/journal.pntd.0007882
32	Sanou, 2018	Clinical and epidemiologic characteristics associated with dengue during and outside the 2016 outbreak identified in health facility-based surveillance in Ouagadougou, Burkina Faso	https://doi.org/10.1371/journal.pntd.00078822
33	Naz, 2013	Evaluation of efficacy of various immunochromatographic rapid tests for dengue diagnosis	https://doi.org/10.12669%2Fpjms.301.4173
34	Krishnananthasivam, 2015	Evaluation of a Commercial Rapid Test Kit for Detection of Acute Dengue Infection	https://pubmed.ncbi.nlm.nih.gov/26867379/
35	Lee, 2015	Enhanced performance of an innovative dengue IgG/IgM rapid diagnostic test using an anti-dengue EDI monoclonal antibody and dengue virus antigen	https://doi.org/10.1038/srep18077
36	Wang, 2010	Early Diagnosis of Dengue Infection Using a Commercial Dengue Duo Rapid Test Kit for the Detection of NS1, IGM, and IGG	https://doi.org/10.4269%2Fajtmh.2010.10-0117
